# Effects of behavioural exercise therapy on the effectiveness of a multidisciplinary rehabilitation for chronic non-specific low back pain: Study protocol for a randomised controlled trial

**DOI:** 10.1186/1471-2474-14-89

**Published:** 2013-03-11

**Authors:** Jana Hofmann, Stefan Peters, Wolfgang Geidl, Christian Hentschke, Klaus Pfeifer

**Affiliations:** 1 Institute of Sport Science and Sport, Friedrich-Alexander-University Erlangen-Nürnberg, Gebbertstr. 123b, D-91058 Erlangen, Germany

**Keywords:** Chronic non-specific low back pain, Exercise therapy, Behavioural exercise therapy, Multidisciplinary rehabilitation, Self-management, Physical activity

## Abstract

**Background:**

In Germany, a multidisciplinary rehabilitation named “behavioural medical rehabilitation” (BMR) is available for treatment of chronic low back pain (clbp). A central component of BMR is standard exercise therapy (SET), which is directed mainly to improve physical fitness. There is a need to address psychosocial factors within SET and therefore to improve behavior change with a focus on the development of self-management skills in dealing with clbp. Furthermore, short-term effectiveness of BMR with a SET has been proven, but the impact of a behavioural exercise therapy (BET) for improvement of the long-term effectiveness of BMR is unclear.

**Methods/design:**

To compare the effectiveness of two exercise programs with different approaches within BMR on the effects of BMR a prospective randomized controlled trial (RCT) in two rehabilitation centres will be performed. 214 patients aged 18–65 with clbp will be, based on an "urn randomisation"-algorithm, randomly assigned to a BMR with SET (function-oriented, n=107) and BMR with BET (behaviour-oriented, n=107). Both exercise programs have a mean duration of 26 hours in three weeks and are delivered by a limited number of not-blinded study therapists in closed groups with six to twelve patients who will be masked regarding study group. The main differences of BET lie in its detailed manualised program with a theory-based, goal-orientated combination of exercise, education and behavioural elements, active participation of patients and consideration of their individual preferences and previous experiences with exercise. The primary outcome is functional ability assessed with the Hannover Functional Ability Questionnaire directly before and after the rehabilitation program, as well as a six and twelve-month follow-up.

**Discussion:**

This RCT is designed to explore the effects of BET on the effectiveness of a BMR compared to a BMR with SET in the management of patients with clbp. Methodological challenges arise from conducting a RCT within routine health care as well as from ensuring high treatment integrity. Findings of this study might contribute to a better understanding of the mechanism of action of BMR and the special effects of BET and may be used to improve the quality of these interventions in routine care, therefore reducing the burden to patients with disabling clbp.

**Trial registration:**

Current controlled trials NCT01666639

## Background

Non-specific low back pain is one of the leading health problems regarding its medical and economic impact worldwide [1-4]. This is also the case in Germany with high substantial economic burden of an estimated €48.96 billion total costs related to low back pain for the entire German adult population aged 18 to 75 [5,6].

While the lifetime prevalence of low back pain is reported to be up to 84%, little scientific evidence is available for the prevalence of disabling chronic low back pain [4,7]. In a multiregional survey in the adult population in Germany reported more than 11% disabling chronic low back pain [5]. Clbp is also one of the most frequent conditions in traditional multidisciplinary inpatient rehabilitation in Germany [8].

Intensive multidisciplinary rehabilitation is recommended as first-line treatment for the management of disabling clbp [4,7,9]. It has been proven to be effective in the short term regarding pain intensity and functional disability compared to no treatment or waiting list controls [10]. But, there is a lack of evidence regarding the long-term effectiveness of such programs. Behavioural medical rehabilitation (BMR) in Germany is an intensive multidisciplinary inpatient rehabilitation program with a theory-based biopsychosocial approach [11]. In contrast to the traditional multidisciplinary inpatient rehabilitation, BMR is explicitly directed at orthopedic patients who show significant psychosocial risk factors for the chronification of musculoskeletal disorders. In a randomised controlled trial, the BMR had a positive short-term effect on depression and sustainable effects on several pain coping competencies [12] compared to traditional inpatient rehabilitation. A meta-analysis of intensified multidisciplinary inpatient rehabilitation for patients with musculoskeletal disorders [13] proved BMR to be superior to usual care regarding a sustainable improvement of the subjective health status. Evidence of long-term effects of BMR on pain and functional disability is lacking. It is also not yet clear which treatment component or combination of treatment components of an intensified rehabilitation regime explains the amount of variance regarding the effects in patients with disabling clbp [14].

A corner-stone is SET, which represents approximately 70% of all interventions within multidisciplinary rehabilitation, received by orthopedic patients in Germany [15]. SET is mainly based on a functional approach with methods to improve physical fitness [15,16]. Evidence shows that SET compared to usual care improves pain and function in patients with clbp, however the reported effect-sizes are low [17,18].

There is also evidence available confirming that psychosocial factors play a pivotal role in the development of clbp [19-21]. Addressing them specifically within exercise therapy might reduce obstacles to recovery and increased function [22,23]. Especially to improve self-management of back pain and the long-term adherence to regular physical activity [24], a broader theory-based approach with appropriate tools and strategies used by physical therapists within SET seems necessary [25,26]. An integration of biopsychosocial education and behavior change as well as the consideration of patients’ individual preferences and the realisation of positive experiences with physical activity seem to be superior to a solely functional approach [17,18,24,26-30].

An enhancement of long-term adherence to physical activity in chronically ill adults via patient-oriented methods directed at motivation and volition (e.g. planning) has been effective [27,31,32]. Conn et al. [27] detected behavior change interventions being effective across medical conditions (effect size 0.45) and showed an average increase in the amount of performed physical activity, which was 48 minutes higher than the increase in the control groups. The Review of Jordan et al. [24] examining interventions to improve exercise adherence in musculoskeletal disorders outlined that adherence is not influenced by the kind of exercise being performed (e.g. strength training, endurance-oriented activities). A combination of exercise with cognitive and behavioural strategies seems to be more important to improve adherence to physical activity or exercise and the addition of volitional methods to motivational methods is paramount [24,27,31-33].

There has not been in-depth research about the biopsychosocial effects of theory-based exercise therapy with a behavioural approach, named behavioural exercise therapy (BET), as part of the BMR in the management of disabling clbp. So the impact of a BET for improvement of the effectiveness of BMR is unclear.

### The aims of this study

Therefore the aims of this study are a) the development and implementation of a BET into an existing BMR and b) the evaluation of the effectiveness of BMR with BET compared to BMR with SET in routine health care (short-, mid-, and long-term effects) in the management of patients with disabling clbp.

## Methods/design

### Study design

**Figure 1:**
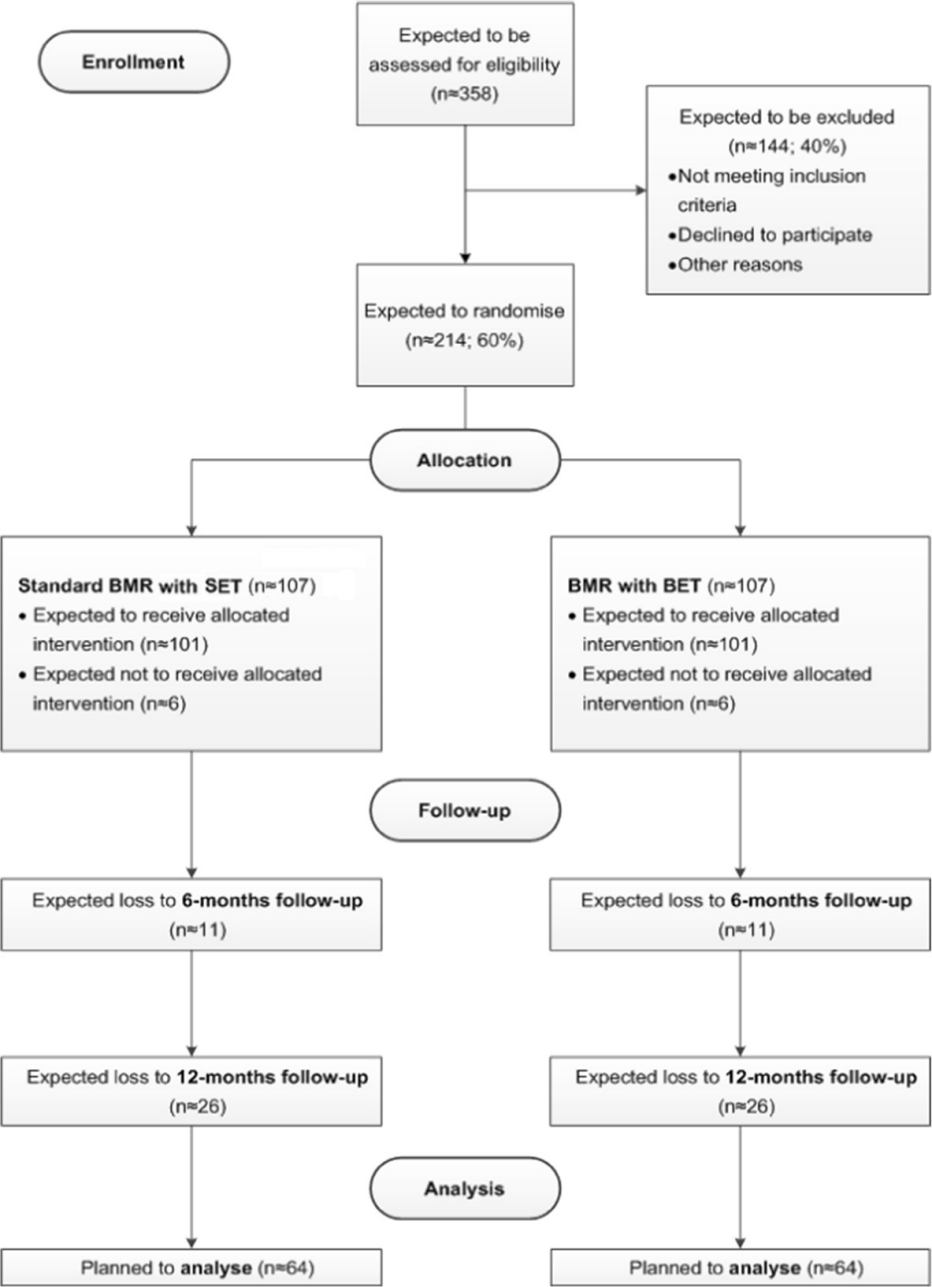
Study Flow Diagram.

**Figure 2:**
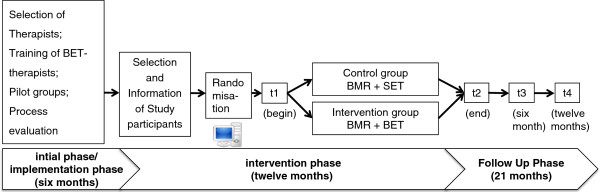
Study phases and expected duration.

To evaluate the effects of BET on the effectiveness of a BMR, a multicentre-study with a prospective randomised controlled design is conducted. This study takes place in two inpatient rehabilitation centres in Germany. Participants with clbp will be randomised into either a) the usual BMR with the SET as the control group (CG) or b) the BMR with the extended BET as the intervention group (IG) (see Figure 1). Primary and secondary outcomes will be measured by questionnaires directly before and at the end of the BMR (after approx. four weeks), as well as six and twelve months after completion of the BMR. The following figure shows the study design with appropriate study phases as well as the expected duration (see Figure 2).

### Hypotheses

#### Primary hypothesis

In patients with clbp, treatment with BMR+BET will result in a significantly higher increase in functional ability directly at the end, as well as six and twelve months after completion of BMR+BET compared to usual BMR+SET.

#### Secondary hypothesis

There are numerous interrelations between self-management of clbp, pain-related cognitions, physical activity, health-related quality of life and the number of back pain episodes [19,21,22,34-37]. For these reasons the following secondary hypotheses are tested:

In patients with clbp, treatment with BMR+BET, compared to usual BMR+SET, will result in a significant:

– Improvement of pain-related cognitions

– Increase of physical activity

– Improvement in health-related quality of life

– Decrease in back pain episodes

– Improvement in pain coping strategies

### Study population

Patients with clbp, who are invited consecutively in the two collaborating rehabilitation centres between January 2012 and January 2013, are eligible by reservation. Chronic non-specific low-back pain is defined as pain persisting for at least three months, localised below the costal margin and above the inferior gluteal folds, without referred leg pain and that is not caused by a known specific pathology [7]. Inclusion criteria are based on the international classification of diseases (ICD-10) in its German modification. Inclusion and exclusion criteria are described in Table 1.

**Table 1:** Inclusion and exclusion criteria

**Inclusion**	**Main diagnosis for a BMR:**
criteria	-M51.2-M51.9 (other intervertebral disc disorders),
(ICD-10)	-M53.8, M53.9 (other specified/unspecified dorsopathies),
	-M54.4-M54.9 (lumbago with sciatica, low back pain, pain in thoracic spine, other/unspecified dorsalgia),
	-F45.4 (persistent somatoform pain disorder),
	-F45.41 (chronic pain disorder with somatic and psychological factors),
	-F54 (psychological and behavioural factors associated with disorders or diseases classified elsewhere)
Exclusion criteria	-specific underlying diagnosis of the back pain (e. g. radicular symptoms, myelopathy)
	-considerably reduced health status (comorbidities)
	-considerable reduction of sight and hearing
	-severe psychiatric condition as secondary diagnosis
	-age below 18 or over 65 respectively
	-lack of ability to speak German
	-ongoing application for retirement

### Recruitment procedures

After submission of the signed consent form of the study participants („informed consent“), patient enrollment takes place in the participating rehabilitation centres based on the assessment of the inclusion and exclusion criteria by the attending physician. Next, patients are randomly assigned to the different treatment groups.

The recruitment procedure is adjusted to each of the centre’s internal structures and processes regarding the invitation of patients.

In the rehabilitation centre “Paracelsus-Klinik an der Gande” potential study participants will be identified by a screening of the patient record by the head physician prior to the invitation to the BMR. Afterwards, eligible patients will obtain written patient information and a consent form about the study and will be asked to participate. After returning the signed consent form within seven days, the patients will be randomised online. The invitation of the randomised patients will take place weekly and in groups according to the group assignment in the respective week of arrival. At the beginning of the rehabilitation program, an information meeting will be held by the head physician for all eligible patients fully explaining the study.

In the rehabilitation centre “Klinik Weser” potential study participants will be identified at the beginning of the BMR in medical admission interviews and will obtain a written consent form. In an information meeting all eligible patients are fully informed about the study primarily by the head physician. On the second day, after returning the signed consent form, the online-based randomisation will take place. Due to spatial and personnel-wise requirements, one control and one intervention group will start simultaneously every fourteen days. Therefore, the recruitment of eligible patients will take place in a biweekly rhythm.

In both rehabilitation centres, the group size is limited to twelve participants. If the number of eligible study patients is too small for starting a control or intervention group, the participation of non-eligible patients who do not take part in the study is accepted to reach the necessary group size. At least 40 potentially eligible patients will be treated every month in both rehabilitation centres. Considering a recruitment rate of 60%, 358 eligible patients will have to be asked to participate in the study in order to achieve the required sample size. Based on the assumption that average closed groups consist of six to seven participants, the recruitment period is predefined as a period of twelve months. Both rehabilitation centres confirmed that a sufficient number of eligible patients would exist to achieve the necessary sample size in the study period.

The collection of consent forms, distribution and collection of questionnaires and the delivery of the latter to the study team are adjusted to the process of patient admission and discharge in standardised form. The process will be tested and automated in each of the rehabilitation centres during the phase of implementation.

#### Follow-up phase

The follow-up questionnaires will be sent to the former participants six and twelve months after the rehabilitation treatment by the university. Postal reminders will be send after three weeks if the questionnaire has not been sent back by patients.

#### Drop out criteria

Dropouts are defined as patients who do refuse to participate in the study at the end of the rehabilitation program. These patients will not be contacted after six and twelve months for further evaluation.

Also, dropouts are patients who withdraw the entire study participation and prohibit the use of existing personnel data. In this case, the questionnaire filled out at the beginning of the rehabilitation program must be given to the patient.

If patients don’t fill out the questionnaire at the end of the rehabilitation (despite former reminder), they will still receive a questionnaire after six and twelve months, if they don’t explicitly refuse or withdrew from the study participation.

#### Registration of non-responders

The registration of all eligible patients and the reporting of the number and the sex of eligible patients who do not participate (non-responder analysis) during the time of the study will take place for the identification of selection effects during the recruitment process.

### Randomisation

The randomisation procedure of the participants takes place with an online-based randomisation feature. Therefore, we implement a central data base for electronic data recording which will be used in each of the two rehabilitation centres. An independent administration employee, who is not involved in the study, will use the online-based randomization feature within each rehabilitation centre to register eligible participants via a web application. After registration, the online-based randomisation feature automatically accomplishes the allocation to one of the treatment groups.This system has been developed in a prior study [38] and incorporates the existing national data protection regulations. Clear advantages of this procedure are a prompt randomisation and a concealment of the sequence of allocation. For sequence generation we use an “urn randomisation”-algorithm [39]. It has good statistical properties [40] and can be used to stratify by different criteria. In this study we stratify for “rehabilitation centre” with the two rehabilitation centres and “gender” with two categories. Additionally, a structural equality by chance concerning both sub-samples is ensured.

The randomisation process of patients will be planned together with the staff of the participating rehabilitation centres, especially the medical directors, administration staff, system administrator and those responsible for therapy planning in the rehabilitation centres during the phase of preparation and implementation. For the planning of the randomisation process, the implementation of the data base and the training of administration staff to handle the data base, the rehabilitation centres will be visit two to three times.

### Sample size and power calculation

Thinking of the short-term effectiveness of both interventions, only small differences between groups and small effect sizes at the end of rehabilitation (t2) (d=0,3 or f(V)=0,15) are expected to be found [12,41,42]. Because of the minor long-term effects of the present usual care, medium effect sizes (d=0,5 or f(V)=0,25) are expected to be found at six and twelve months. This is in line with Friedrich and colleagues [43,44], who were able to show medium effect sizes regarding a reduction of disability when comparing a functional exercise therapy to an exercise therapy which contained additional motivational strategies (Disability Score: d~0,68). Sample power (Software: G-Power 3.0) concerning the primary outcome was approximated for an analysis of covariance at t4 with medium effect size of Cohen’s d=0,5, alpha error 5% and test power of 80%. This approximisation results in a sample size of at least 128 patients without missing values. Anticipating a dropout rate of 40%, it is necessary to include 214 patients (n=107 for each group).

### Interventions

#### Control arm

Both of the participating rehabilitation centres have a conceptually similar BMR for patients with orthopedic functional impairment and concurrent substantial psychological or social component of this impairment. The behavioural medical concept takes into account the biopsychosocial mechanisms relevant for the chronification of musculoskeletal disorders [9,45]. Empowerment and self-management are encouraged. On average, the BMR lasts for 27 days. Compared to the traditional multidisciplinary inpatient rehabilitation, the BMR has specifically defined inclusion criteria [11]: 1) an interdisciplinary admission, 2) a standardized psychosocial assessment, 3) a reconciled interdisciplinary case management, 4) case reviews on a regular basis, 5) supervision, 6) closed groups (a group remains together in all treatments during their whole stay at the rehabilitation centre), 7) the possibility of an individually tailored rehabilitation schedule and 8) therapist consistency for the most part (a therapist accompanies a group throughout a whole intervention). The multiprofessional core of the BMR relies on psychological interventions and standard exercise therapy (SET), in which ten to twelve patients take part together as a closed group. Other interventions are presentations with information about health and health behavior, rehabilitation/social counseling and if necessary occupational therapy. The total extent of therapy during the stay in the rehab centre is 65 hours on average, including approximately 26 hours exercise therapy. An experienced orthopedist bears the overall responsibility. A detailed description of the psychological group and exercise therapy group is available in the Additional files (see Additional file 1).

#### Intervention arm

Participants in the intervention arm will also receive a BMR, but with a specific behaviour-oriented exercise therapy (BET), which replaces SET. BET has been developed on the basis of previous work [46]. In general, BET aims at facilitating health-related knowledge and skills, which empower patients to maintain a healthy, physically active lifestyle beyond their BMR treatment. It’s based on the assumption that a theory-based, systematic and integrated targeting of relevant psychosocial factors in the chronification of low back pain and also determinants of health behavior change, as well as factors of physical deconditioning and disuse improves the self-management and therefore reduces functional impairment of patients with clbp. This is reached by a structured combination of exercise, education and behavioural elements with a strong emphasis on patients’ preferences and their previous experiences with exercise. Therefore different types of exercise are considered for patients to become familiar with their execution (e. g. methods of controlling intensities and training progression), as well as to encourage positive experiences with exercise. BET is delivered in closed groups with six to twelve patients during the approximately four weeks of the BMR. In total, exercise therapy comprised an extent of 26 hours on average. Both the fifteen sessions and the related modules of BET are guided by the same trained BET-therapist.

#### 15 BET sessions, 60 minutes duration

The major part of BET consists of fifteen sessions with duration of 60 minutes each. Every single session comprises an introduction (approx. 5min), structured active play (approx. 5min), short education periods on selected topics (approx. 10min), exercises and relaxation (approx. 30min) as well as conclusion and sporadically a metaphoric story (approx. 10min). The entire thematic topic overview of the fifteen sessions can be seen in the in the Additional files (see Additional file 2).

#### BET related modules, 20 to 60 minutes duration

In the first week patients receive, two 60 min. introductions in weight-lifting training, two 60 min. introductions in an aerobic exercise (walking, nordic walking and ergometer cycling) and two 30 min. introductions in aqua training (aqua aerobics, aqua jogging). Further three modules of planning out of every 60 minutes duration are distributed over three weeks. The purpose of these related modules is for patients to become familiar with different types of exercises in the first week supervised by a BET-therapist followed by a more self-directed planning and performing of exercises during the rehabilitation stay. Therefore during the four weeks of treatment the supervision by the therapist decreases, while the performing of self-directed exercises increases. At the end of the second week, the BET-therapist will reflect together with the group reviewing, how well the individual realisation of planned exercises itself is managed. If barriers occur, they will be discussed and alternative possibilities will be developed. To be available for consult in case of any questions especially related to weight-lifting training, additional three appointments will be scheduled (one each in the 2nd to 4th week of therapy) under supervision of a BET therapist. Table 2 shows the volume of related modules with duration, frequency and distribution over the four weeks of the rehabilitation phase.

**Table 2:** BET related modules

**BET related modules**	**Duration**	**Frequency/ week of rehabilitation**
Weight-lifting training introduction	60min	2x/ week 1
Weight-lifting training	60min	3x distributes over week 2 to 4
Aerobic exercise introduction	60min	2x/ week 1
Aquatic training introduction	30min	2x/ week 1
Planning module	60min	3x distributes over week 1 and 2
Individual physical activity/ exercise	----	self-directed physical activity/ exercise distributes over week 2 to 4

In summary, the following key features characterized BET:

– Patient-centred approach through active participation of patients in the entire treatment process and considering their individual preferences and previous experiences with exercise

– Goal-oriented combination of exercise, education, and behavioural elements within the BET

– Psychosocial factors that contribute to the chronification of clbp (e. g. pain-related cognitions, avoidance behavior, endurance behaviour) are systematically modified by the use of structured contents and methods which refer to exercise, education and behavioural elements

– Theory-based use of behavioural techniques to influence motivational and volitional determinants of health behavior change to promote long-term maintenance of self-directed physical activity

– Specific patient-oriented, plain media and materials with a very demanding character are used

– Trainer manual with a detailed description of the BET program for a standardized execution of the exercise, educational and behavioural elements, as well as a standardized use of media and materials to achieve multidimensional objectives of the BET is available

– Objectives, contents and methods of BET are matched in terms of a consistent approach and cooperation of all professions involved in the BMR

– Key messages and competencies trained by medical and psychological professions in the BMR will be reflected and reinforced within the BET.

More information can be found in the Additional files (see Additional file 3) and the detailed trainer manual is available in German and can be requested from the authors.

#### Treatment adherence

The therapists who perform BET in the intervention group will receive an intensive training on the objectives, contents, structure, components and methods of the program. During the implementation phase two workshops (each 2 days with 720 min duration) at intervals of eight weeks will take place together with all BET-therapists from both rehabilitation centres. The workshops are provided on the basis of a manualised intervention concept and are delivered by the same trainers. Moreover, pilot groups with the BET program will be performed within the implementation phase. The BET-therapists are skilled in the use of the program and try out the approach with the specific goal-orientated components as well as the media and materials.

The intensive training of BET-therapists and the implementation of the BET-groups will be supervised and evaluated. For data collection, quantitative methods are used (self-constructed questionnaires). The acceptance of the therapists (motivation, readiness to change, and program satisfaction) and difficulties during the implementation as well as acceptance of the patients (program satisfaction) will be recorded.

Both exercise programs (SET and BET) within the BMR have the same duration. The duration of both exercise programs as well as additional interventions within the BMR are documented for each study patient during the intervention phase.

A potential source of bias in the execution of RCT in the inpatient rehabilitation is the potential mixture between the study group and the control group through a communication about intervention contents between patients as well as therapists within each rehabilitation centre. The adaptations in the BET aimed at the integration of behavioural techniques and a modified use of teaching approaches. A reflected effective exchange of the adapted intervention contents requires extensive knowledge of the underlying theoretical models of the chronification of lbp. This is generally not expected from patients of a BMR. In addition, the patients of both study groups will be informed that both exercise programs within in the BMR are of high quality and will be delivered based on actual scientific evidence.

Trained BET-therapists will sign a written contract concerning discretion about objectives, contents, structure, methods, media and materials used within the BET. Regularly, announced visitations during the intervention phase will occur in order to assure adherence to the treatment protocol. Individual deviations from the treatment protocol will be recorded. In the case of vacation or sick leave, a trained BET-therapist for replacement is available.

### Outcome assessment

Standardised questionnaires will be used to measure the primary and secondary outcomes. Our choice of outcome measures is based on two previous trials of our working group to evaluate effectiveness of different interventions in the management of clbp [38,46]. They are also in line with international recommendations [47-50]. All outcomes measures and their measuring time are shown in Table 3.

**Table 3:** Primary and secondary outcomes

**Domain/outcome**	**Questionnaire/items**	**Assessment time point**				**Reference**
**Primary Outcome**		t1	t2	t3	t4	
**Physical functioning**	Hannover Functional Ability Questionnaire (FfbH-R)	x	x	x	x	[51]
**Secondary Outcomes**						
**Pain**						
Pain intensity	Numeric Rating Scale (NRS)	x	x	x	x	[52]
Pain status	Graded Chronic Pain Status (GCPS)	x	x	x	x	[53]
**Physical activity**	Freiburg questionnaire on physical activity (FFkA)	x		x	x	[54]
**Emotional functioning**						
Depression	Patient Health Questionnaire (PHQ-D)	x	x	x	x	[55]
Anxiety	Generalized anxiety disorder 7-item scale (GAD-7)	x	x	x	x	[56]
**Health related quality of life**	Short-Form-12 (SF-12)	x	x	x	x	[57]
**Stress**	Perceived Stress Scale (PSS)	x	x	x	x	[58]
**Pain related cognitions, emotions and behavior**						
Cognitive and behavioral pain coping strategies	Pain Management Questionnaire (FESV)	x	x	x	x	[59]
Fear-avoidance and endurance-related responses to pain	Avoidance-Endurance Questionnaire (AEQ)	x	x	x	x	[60]
Pain related fear	Tampa Scale for Kinesiophobia (TSK)	x	x	x	x	[61,62]
**Motivational and volitional determinants of physical activity**						
	risk perception (3 items), self-efficacy (3 items), outcome expectancies (11items), experiences with physical activity (5 items), self-concordance (12 items), intention (7 items), action (4 items) and coping (4 items) planning, action control (6 items)	x	x	x	x	[63-67]
Stage of change algorithm for physical activity	two Items (Have you performed moderate physical for 30 minutes or longer on a minimum of 3 days per week? and "Since when are you as regularly active as you are now?"; possible answers: 1. Question: no, and I don’t intend to do so; no, but I am thinking about doing so; no, but I have the strong intention to do so; yes, but it is difficult to me; yes, and it is easy for me; 2. Question: open)	x	x	x	x	[68,69]
**Attitudes towards performing physical activity**	modified Version of Short-Questionnaire to measure cognitive and affective attitudes toward sports activities	x	x	x	x	[70]
**Treatment satisfactions with exercise therapy**	18 items (e.g. “I would recommend the exercise therapy other patients.” measured by using a six-point ordinal scale with 1= “absolutely agree”, 6=“absolutely disagree”; e.g. “The time extent of the exercise therapy, I perceived as…” measured by using a five-point ordinal scale with 1=“far too high“, 5=“far too low“; e.g. “Overall, how do you rate the exercise therapy?” measured by using a six-point ordinal scale with 1= “very satisfied”, 6=not “satisfied at all”)		x			self-designed
**Treatment satisfactions with the rehabilitation process**	five items (e.g. “Overall, how do you rate the rehabilitation process?”) measured by using a ten-point ordinal scale (1= “very poor”, 10 = „excellent“)		x			self-designed
**Others**						
Demographic characteristics	sex, age, height, nationality, marital status, education, monthly income, weight, self-reported work status	x				[71]
Social medical characteristics	a) self-reported low back pain related sick leave, use of health care services, medication use for the last six month and self-reported plan to apply for retirement, severe disabilities	x				[71]
	b) diagnosis, work status and physical capability taken from the hospital discharge report		x			
Aftercare	a) aftercare recommendations taken from the hospital discharge report		x			self-designed
	b) self-designed; five items (e.g. “Have you participated in the last twelve months in a medically prescribed exercise therapy to rehabilitation aftercare?” with the option to answer yes/no)				x	
Job satisfaction	scale on job satisfaction (IRES-3)			x	x	[72]

#### Primary outcome measure

We will use the level of functional limitations associated to clbp twelve months after the end of BMR measured by the Hannover Functional Ability Questionnaire (FFbH-R) as our primary outcome [51]. The FFbH-R consists of twelve items with a three-stage answering scale. Its summary score describes the low back pain associated functional ability in activities of daily living in adults on a scale of 0% (minimum functional ability) to 100% (maximum functional ability). The questionnaire is constructed for response to already light and moderate functional restrictions. The average item intercorrelation amounts to 0.50. The test-retest-reliability with repeated measures after approximately one week is above 0.75, Cronbach alpha figures 0.90 [51]. The one factorial structure of the instrument was confirmed in a principal component analysis. The FFbH-R is comparable to the internationally used Roland Morris Questionnaire for measuring clbp related disability with a correlation of 0.75 [73].

#### Secondary outcome measures

Secondary outcome measures refer to demographics and related characteristics, pain, physical activity, health-related quality of life, depression, anxiety, stress, pain-related cognitions, emotions and behavior, motivational and volitional determinants of physical activity, attitudes towards performing physical activity, satisfaction with exercise therapy and satisfaction with the rehabilitation process (see Table 3).

### Data analysis

Data analysis is carried out following evaluation standards of experimental study designs with control groups, using descriptive methods as well as inferential statistics. An intention-to-treat analysis will be performed. In the case of dropouts and withdrawals, multiple imputation techniques will be used. Baseline differences in demographic as well as the primary and secondary outcomes data will be analysed using two-sample t-tests for parametric and Wilcoxon test for non-parametric distribution as well as Chi-Square test for nominal data. The testing of the hypotheses is conducted via comparison of changes in the intervention and the control group regarding the primary and secondary outcomes. For this purpose, a saturated 4x2-factorial linear mixed effects model in the process of all four assessment time points and two groups as fixed effects and intercept and slope as random effects is used whilst controlling for statistically significant differences at baseline [74]. Changes in outcomes over time are represented by their linear slopes. Because of the disparity between the duration of the inpatient rehabilitation phase (27 days on average) and the follow-up phase (12 months), separate slopes for these phases are estimated by a spline model. Primarily overall group differences are identified by a model comparing likehood ratio test of a hypothesized model incorporating interaction effects of group by time and a nested null model leaving out these interactions. Additionally the slopes of both groups can be compared in two phases whilst controlling for statistically significant differences at baseline. Furthermore, a post hoc analysis using similar mixed effects linear regression techniques is conducted to model effects of secondary outcomes onto the primary outcome to explore possible interactions.

### Blinding

Blinding of the therapists is not possible because they will be trained to perform the BET or will be chosen deliver the existing exercise therapy within the BMR during the study period.

Patients will be masked regarding the study group. They will be informed through the staff and in the “informed consent” that the effectiveness of two exercise therapy programs will be compared and that both meet current scientific standards and are appropriate to improve health status. During the study period, patients will be not informed as to whether they participate in the control group or intervention group.

A person who will not be involved in the study process performs the statistical data analysis. Furthermore, the evaluation will be blinded to the treatment group.

### Ethical aspects

Ethical and legal problems are not anticipated because of experiences with comparable studies. This investigation is conducted according to the recommendations of the World Medical Association (Declaration of Helsinki: [75]). Information for the participants and their agreement on study participation is written down as „informed consent“. It is pointed out to the participants that the study participation is voluntary and that they may refuse to participate or to discontinue participation at any time without disadvantages or loss of benefits. According to the national data protection laws all personal data is treated as confidential and is used only for scientific purposes. Ethical approval has been granted by the independent Research Ethics Committee of the Medical Faculty of Friedrich-Alexander-University of Erlangen-Nürnberg (Re.-No. 4510). High ethical demands are also imposed by the study sponsor, the German Pension Insurance Association (Deutsche Rentenversicherung Bund). There is a special focus on data privacy.

## Discussion

In the management of disabling clbp intensive multidisciplinary rehabilitation is recommended. But, the reported effects especially in the long-term are low [8,10]. A traditional approach of exercise therapy which is often deduced from physiological models is a cornerstone within multidisciplinary rehabilitation. With BMR, an intensified and theory-based rehabilitation program with proven short-term effectiveness is available in Germany [12]. Exercise-therapy is a central component within BMR, but in practice a biomedical approach mainly to improve physical fitness has established which might work as an obstacle to enhance the long-term effects of a BMR. However, the growing empirical evidence for the importance of psychosocial factors in the development of clbp [19] and the weak relationship between changes in physical function measures and changes in various health outcomes in clbp after exercise therapy [76] requires a more behavioural approach of exercise therapy to facilitate self-management and adherence to regular physical activity and therefore reducing pain and disability in the long-term [22]. Only two exercise therapy programs with a theory-based and behavioural approach are available for patients with clbp in Germany, both with a focus to enhance exercise adherence [31,32]. The majority of exercise therapy programs in the routine of traditional multidisciplinary inpatient rehabilitation in Germany are not explicitly theory-based, so it is unclear which factors of the chronification process and causal mechanism of clbp are influenced. On the other hand, causal mechanisms remain inadequately clarified, even in theoretical models for clbp [20,77,78]. The evaluation of theory-based exercise therapy programs can contribute to the further development of theoretical assumptions of the chronification process of clbp.

Therefore, there is a need for the development, implementation and evaluation of a theory-based exercise therapy with a behavioural approach (BET) in the context of a BMR. To our knowledge, this is the first RCT to examine the effects of BET for the overall treatment success of BMR in the routine healthcare management of patients with disabling clbp.

Methodological challenges arise from conducting a RCT, which claims a lot of resources, within routine health care in two rehabilitation centres as well as from ensuring high treatment integrity. The latter refers particularly to a potential mixture between the study group and the control group through a communication about intervention contents between patients and therapists within each rehabilitation centre, as well as individual deviations from the treatment protocol. To overcome those potential sources of bias BET is delivered by the same BET-therapist, who will be intensively trained in the use of the program and will receive a detailed trainer manual. BET-therapists will sign a written contract concerning discretion about BET. Regularly, announced visitations during the intervention phase will occur in order to assure adherence to the treatment protocol. Individual deviations from the treatment protocol will be recorded.

Altogether, findings of this study might contribute to a better understanding of the mechanism of action of BMR and the special effects of BET and might be used to improve the quality of these interventions in routine care and therefore reduce the burden to patients with clbp.

## Abbreviations

BMR: Behavioural medical rehabilitation; BET: Behavioural exercise therapy; clbp: Chronic non-specific low back pain; min: Minutes; RCT: Randomised controlled trial; SET: Standard exercise therapy.

## Competing interests

The authors declare to have no competing interests.

## Authors’ contributions

JH drafted the manuscript and contributed substantially to the development of the research questions, the study design and the study protocol in general. Prior to all of that, JH was also responsible for conducting an in-depth literature search to prepare the BET and the study as a whole. JH is further responsible for the study organisation and oversees patient enrollment and data management. JH is also the coach at the train-the-trainer seminar, who teaches the main part. SP contributed to the development of the assessment tools for the research questions and was a major contributor to the creation of the BET and also teaches several parts at the above mentioned train-the-trainer seminar. SP wrote the respective chapters of this manuscript and contributed substantially to the other chapters. CH contributed substantially to the determination of primary and secondary outcomes and the according assessment tools. CH further oversaw the creation of the study design and is the main contributor regarding randomization, data management and data analysis. WG contributed substantially to the development of the research questions, the study design and the development of contents, methods and materials of BET. KP conceived the study and was responsible for identifying the research question beforehand. KP drafted the study design and was accountable for determining primary and secondary outcomes. KP contrived the basis for development of the experimental treatment. Every author read and approved the final version of this paper.

## Pre-publication history

The pre-publication history for this paper can be accessed here:

http://www.biomedcentral.com/1471-2474/14/89/prepub

## Supplementary Material

Additional file 1 Description of two main interventions within BMR - psychological group therapy and standard exercise therapy (SET) in both participating rehabilitation centres (control group). Click here for file

Additional file 2 Intervention group. Click here for file

Additional file 3 BET_Therapy plan. Click here for file
