# Supplementary material for: Effects of behavioural exercise therapy on the effectiveness of a multidisciplinary rehabilitation for chronic non-specific low back pain: Study protocol for a randomised controlled trial
Source: BMC Musculoskelet Disord. 2013 Mar 11;14:89. doi: 10.1186/1471-2474-14-89 (PMC3610103; doi:10.1186/1471-2474-14-89)
Supplement: Additional file 1 — Description of two main interventions within BMR - psychological group therapy and standard exercise therapy (SET) in both participating rehabilitation centres (control group). [file 1471-2474-14-89-S1.pdf]

Additional file 1: Description of two main interventions within BMR - psychological group therapy and standard exercise therapy (SET) in both participating rehabilitation centres (control group)

	REHABILITATION CENTER 1	REHABILITATION CENTER 2
	psychological group therapy	
theoretical approach	- cognitive-behavioural approach	- cognitive-behavioural approach
frequency/ duration	- six sessions each 60 minutes	- eight sessions each 60 minutes
delivery	- closed group<br>- consistency of the therapist	- closed group<br>- consistency of the therapist
objectives and contents	- introduction to the multimodal therapeutic approach of the BMR<br>- the instruction of a biopsychosocial approach regarding the chronification of pain<br>- the instruction of the avoidance-endurance-model, techniques to control attention,<br>- emergence of a memory of pain<br>- how to use enjoyment and pleasure as a resource,<br>- self-care<br>- helpful thoughts when coping with pain<br>- relationships between pain and social competence<br>- self-management is encouraged with techniques such as homework after the course	- introduction to the multimodal therapeutic approach of the BMR,<br>- education about the biopsychosocial model regarding a chronification of pain<br>- pain acceptance<br>- the role of thoughts and feelings/emotions<br>- the perception of pleasure and enjoyment<br>- pain-related behaviour<br>- sleep hygiene<br>- social competence<br>- excessive demand and retreat<br>- the development of goals for every day life
standardization	- manualised<br>- handouts for patient	- manualised<br>- handouts for patient
additional interventions	- progressive muscle relaxation<br>- opportunity of regular individual visits of the psychotherapist	- progressive muscle relaxation<br>- Feldenkrais<br>- opportunity of regular individual visits of the psychotherapist
	standard exercise therapy	
theoretical approach	- mainly somatic-functional approach	- mainly somatic-functional approach
objectives	- physical conditioning via mobilisation and strengthening of the muscles<br>- improvement of cardiopulmonal endurance,<br>- a reduction of pain as result of good postures,<br>- introduction of relief-postures,<br>- education about (patho-) physiological relation as an improvement in well-being due to a better body awareness	- improvement of physical capability,<br>- reduction of endurance- and avoidance behaviour,<br>- improvement of one's body perception and with existing pain,<br>- testing of one's movement boundaries and the re-gain of confidence in one's own capability<br>- water-based exercise is dedicated to a reduction of pain via unloading and the experience of an improved flexibility in the water
mean duration	- approx. 26 hours in four weeks	- approx. 26 hours in four weeks

delivery	closed group<br>consistency of physical therapist	- closed group<br>- consistency of physical therapist
contents (frequency/ duration)	- strengthening, stabilizing, coordination and flexibility exercises (5 sessions/30 minutes)<br>- introduction to weight-lifting training (2 sessions/30 minutes)<br>- weight-lifting training (5 sessions/60 minutes)<br>- back school consisting of education about anatomy and physiology of the spine, causes of clbp and body mechanics for daily activities (3 sessions/60 minutes)	- strengthening, stabilizing, coordination and flexibility exercises<br>- education about muscular imbalances and the anatomy of the spine<br>- good or healthy posture in every day life: when sitting, standing, getting up, laying down, lifting and carrying objects<br>(content of 5 sessions/45 minutes and seven sessions/30 minutes)<br>- water-based therapy (10 sessions/30 minutes)<br>- instruction to weight-lifting training (2 sessions/45 minutes)<br>- weight-lifting training (6 sessions/30 minutes)<br>- cycling on an ergo-bike (6 sessions/30 minutes)<br>- assessment of physical capability in the beginning of the BMR (1 sessions/100 minutes) and at the end of it (1 sessions/ 60 minutes) with a functional test
standardization	manualized<br>leaflet with strengthening exercises for the back<br>take-home charts with the strengthening exercises to foster long-term adherence after rehabilitation<br>training schedules	- manualized<br>- take-home handout with exercises<br>- training schedules
additional interventions* (*delivered in open groups)	introduction to walking (1 session/30 minutes)<br>walking, or cycling on an ergo-bike (7-10 sessions/90 minutes)<br>water-based exercise (6 sessions/30 minutes)	- introduction to (nordic-)walking (1 session/45 minutes)<br>- (nordic-)walking (5-9 sessions/60 minutes)
