# Supplementary material for: Effects of behavioural exercise therapy on the effectiveness of a multidisciplinary rehabilitation for chronic non-specific low back pain: Study protocol for a randomised controlled trial
Source: BMC Musculoskelet Disord. 2013 Mar 11;14:89. doi: 10.1186/1471-2474-14-89 (PMC3610103; doi:10.1186/1471-2474-14-89)
Supplement: Additional file 2 — Intervention group. [file 1471-2474-14-89-S2.pdf]

Description of the BET (intervention group)

Theoretical foundations

Theoretical foundations of BET are a) cognitive-behavioural models of the chronification of back pain as the fear-avoidance model [1] and the avoidance-endurance model [2], b) theories of health behaviour change [3] such as the health action process approach [4] and c) evidence about the effectiveness of exercise, patient education and cognitive-behavioural interventions in the treatment of clbp [5-7].

The complex mechanisms behind the effects of intensified multidisciplinary rehabilitation (such as the BMR) in the treatment of strongly disabling clbp are not untangled so far. Exercise therapy in such rehabilitation interventions is often primarily used for the purpose of reducing deconditioning, which could reduce pain and disability in the long run [8]. BET relies on the assumption that a goal-oriented integration of elements from exercise therapy, as well as educational and cognitive-behavioural approaches systematically leads to additional psychological effects in addition to the physiological effects. This might result in an even greater reduction of pain and disability as well as in sustainable improvements of patients‘ self-management of their clbp. In detail, elements of BET should have a positive influence on mood, maladaptive pain-related cognitions (e. g. fear-avoidance beliefs, catastrophizing) and determinants of health behaviour change which all together may influence pain and disability.

General objectives and subordinate goals

The theoretical background and the existing evidence of the effectiveness of exercise, patient education and cognitive-behavioural interventions lead to three more general objectives of the BET: 1) The development of active self-management strategies when coping with clbp, 2) The introduction to physical activity and the long-term maintenance of the latter and 3) The improvement of health-related fitness. To achieve the general objectives, appropriate elements of exercise are systematically combined with patient education and behavioural techniques. Figure 1 provides an overview of the subordinate goals behind the general objectives.

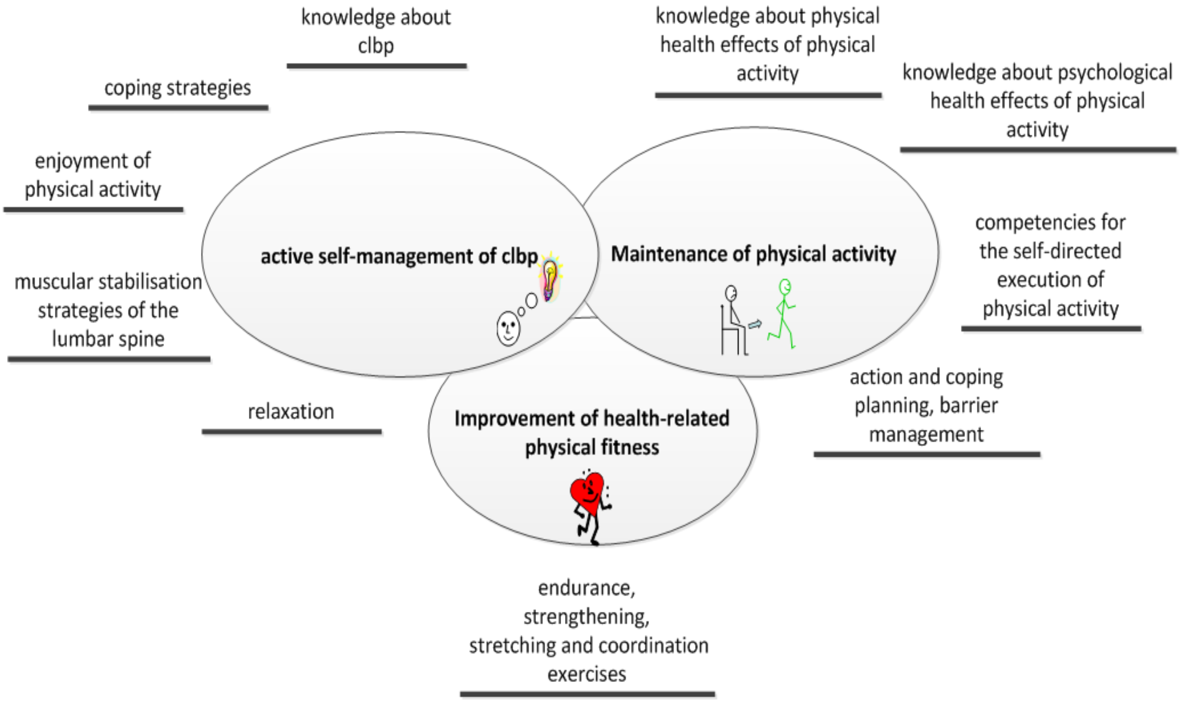

Figure 1: Overview of the general objectives of BET and the subordinated goals

Exercise, patient education and cognitive-behavioural techniques

Table “therapy plan” in Additional file 2 (see add2_BET_therapy plan) outlines the content for exercise (light blue), the facilitation of education about clbp (green) and health effects of physical activity (yellow) as well as the facilitation of coping strategies (purple). Especially in the parts “education about clbp” and “coping strategies”, information which was already provided in other therapies of the BMR (e.g. in the psychological group therapy), are repeated and intensified. This happens for the purpose of having a common message in the whole team of the BMR, which provides patients with a consistent depiction of clbp und reduces uncertainty due to incoherent messages from different professions or therapists. The direct connection of such content with exercise interventions most likely leads to an intensified influence of pain-related factors, as they are described in the cognitive-behavioural models of chronification. BET comprises the following contextual and methodological core themes:

- The focus is not on becoming free of pain, but of a changed behaviour in dealing with clbp
- BET-therapists are educated to support adaptive behavior with encouragement as opposed to maladaptive behaviour (e.g. respective

mimics, gestics, posture and other showings of pain), which is mainly to be ignored

- Anatomy and function of the spine are provided with a positive focus on its highly developed capacity and its elaborated function
- Education about the positive physiological health-related effects of physical activity and its psychological benefits (e.g. stress and mood management) takes place
- The relevance of maladaptive pain-related cognitions and behaviour (e.g. avoidance or endurance behaviour), as well as adaptive cognitions and behaviour (e.g. pa for relaxation or pleasure) is brought up
- Positive experiences and pleasure regarding exercise and physical activity are fostered
- Active strategies of muscular stabilization of the spine when working or doing activities of daily living are introduced, as well as movements to compensate for very monotonous postures
- Body perception and perception of exertion are trained
- Movement and regulation competencies for self-directed physical activity with the support of subjective and objective methods are introduced

Type of exercise and training principles

Evidence for superior effectiveness of any type of specific exercise over another in the management of clbp is sparse [5, 9]. Currently, there is no evidence for superior effectiveness of specific types of exercises based on the fear-avoidance models such as graded-activity and exposure in vivo in the management of clbp [10, 11]. It is possibly more important to consider patient's experiences and individual preferences in the choice of exercise and to encourage positive experiences with exercise [12] Accordingly, the BET includes different types of exercise such as aerobic exercise, weight-lifting training, strengthening exercise, stabilization exercises, movement play, stretching exercises, aqua training and alternative forms of exercise such as qi gong. In the delivery of different types of exercise there is a major focus on a self-directed realisation of exercises. For example, within the first three of the fifteen sessions a functional gymnastic program consisting of five exercises is introduced to the patients guided by the BET-therapist. Patients carry out these exercises as standard program in the further sessions in a self-directed manner. Furthermore, within the additional

modules patients become familiar with different types of exercise guided by the BET-therapist and then select an exercise that they individually plan and realise during the rehabilitation stay. There is a lack of evidence for a specific dose-response relationship in exercise therapy in the management of clbp. Therefore specific training principles cannot be derived. They depend on individual physical capacity and individual desired goals of the patients. For the BET, training principles are guided by the recommendations of the World Health Organisation (WHO), the American College of Sports Medicine (ACSM) and the American Heart Association (AHA) for health-enhancing physical activity [13, 14]. The progression of training dosage within in the BET is used instead of a quota system, based on the subjective perceived exertion by use of the “Rate of Perceived Exertion”-Scale (Borg scale) ranging from 6–20. Individuals will be encouraged to exercise at a level that is ‘somewhat hard’ [15]. Additionally, patients are also skilled in manual pulse measurement to control intensities of aerobic exercises.

Behavioural techniques of behaviour change

In a previous work of our working group in cooperation with the working group “exercise therapy” of the German Society of Rehabilitation Science (DGRW), we derived motivational and volitional determinants from theories of health behavior change [16]. Afterwards we identified effective techniques for enhancing physical activity based on an international review [17] and control-group studies from the German rehabilitation setting [18-21]. This resulted in a list of 28 techniques, of which 22 are used in the fifteen units and the additional modules of the current BET-program (see table 1).

Table 1: motivational and volitional techniques of behaviour change used within BET (Geidl et al. 2012)

Nb.	Techniques with a motivational focus	Short description of the technique	Determinants
1	Generate own varied activity experiences	Get to know and try out different forms of PA	Self-efficacy, self-concordance
2	Information about the execution of health-enhancing physical activities	Information about, e.g. methods of controlling intensities, optimal execution of sports-related exercise techniques etc. while taking into account indication-specific characteristics	Knowledge, self-efficacy
3	Information about the optimal dose of PA	Indication-specific recommendations for health-enhancing PA and sports	Knowledge, outcome expectation
4	Information about consequences of physical (in-) activity	Communicate the negative consequences of lack of PA respectively the positive impact of regular PA and the relevance for functioning, disability and the course of the disease	Risk perception, knowledge, outcome expectation
5	Appropriate level of difficulty of exercise programs	Design programs that are not too easy and not to demanding, where patients can successfully accomplish the tasks and where they can trace back the success to their own abilities (including a personalized exercise plan with intensities, durations, progression etc.)	Self-efficacy
6	Provide feedback about the current exercise performance	Precise positive feedback about exercise performance or evaluation of performance in comparison to a defined standard or other participants	Self-efficacy, outcome expectation, outcome experience
7	Use of attention instructions/ perception guidance	Draw attention to the perception of physiological changes during the accomplishment of exercise related situations and work towards a positive/appropriate interpretation of occurring body reactions	Self-efficacy, outcome expectation, outcome experience
8	Mood management	Support the self evaluation of the emotional status before and after an PA	Outcome expectation, outcome experience
9	Clarify the relevance of training methods	Information about the relevance of different exercises for activities of daily living	Knowledge, outcome expectation
10	Convey information about the execution of activities of daily living	Information about how to execute activities of daily living with impairments, e.g. climb the stairs with a hip-prosthesis	Knowledge, outcome expectation
11	Cost-benefit analysis / decision balance	Contrast costs and benefits of regular PA; reduce and mitigate/moderate vs. negative expectations of taking up PA	Goal, outcome expectation
12	Reduction/moderation of negative experience of	Addressing of negative experiences related to exercise and PA and development of solutions for the causes of the problems	Self-efficacy, goal,outcome expectation, outcome

	consequences		experience
13	Secure/assure positive experiences of consequences	Discussion of the satisfaction with the achieved consequences of PA based on the beforehand formulated realistic objectives and expectations	Outcome expectation, outcome experience
14	Framing activity goals	Concrete formulation and written confirmation of personal, short-term, achievable but appropriate exercise goals, especially for the time after the rehabilitation	Implementation planning, self-efficacy
15	Examine the self-concordance	The self-created exercise goals have to be reflected according to the personal interests and values	Self-concordance
16	Monitor changes of physical functioning	Repeated measurement of physical functioning, e.g. with a fitness test, with explicit documentation and reflection about the improvements	Self-efficacy, outcome expectation, outcome experience
	**Techniques with a volitional focus**	**Short description of the technique**	**Determinants**
17	Information about possibilities to continue back home	The patients get concrete information about possibilities to stay physically active within organized exercise programs or independent self-regulated activities after rehabilitation	Knowledge, implementation planning
18	Development of implementation plans	Formulation of concrete exercise plans for the attainment of the activity goals after rehabilitation through the specification of the “w”-questions (what, when, where and with whom)	Implementation planning
19	Identify barriers	Individual identification of barriers for unorganized PA or the participation in organized exercise programs	Coping strategies
20	Formulation of coping strategies	Development of appropriate counter strategies for the determined barriers	Coping strategies
21	Instruction and use of self-monitoring	Practice and usage of self-monitoring materials (exercise diaries, training documentation, step counter). Comparison of the self-formulated goals with the actual behaviour or the training improvements	Action control
22	Relapse prophylaxis	Addressing of relapses into a physically inactive lifestyle and possibilities of handling relapses	Coping strategies, action control, self-efficacy

Educative format and teaching approaches

Due to the integrative combination of exercise, patient education and cognitive-behavioural interventions, an interactive education format is used with different teaching approaches. These include interactive short presentations and moderated group discussions with the use of flip charts and take-home cards for knowledge transfer, demonstration or verbal instructions by the BET-therapist, individual practice or group exercises, monitoring tasks and attention shift, connecting cognitive and motor learning processes, as well as perception of tension and relaxation of muscles. Patients always have the opportunity to ask questions and to describe their own experiences.

Trainer manual

BET is described thoroughly in a manual in very detail (see table 2) and includes specific short overviews, components and exercise collections, which can be assigned to the above-mentioned general objectives.

Table 2: Overview about the content of the trainer manual

Content of the trainer manual	Number
Short overviews of the fifteen sessions	15
Components of the fifteen sessions	96
Components of the additional module strength training	6
Components of the additional module aerobic exercise	14
Components of the additional module planning	6
Cards for patients	59
Media for BET-therapists (flip chart)	6
Media for BET-therapists (DVD)	1
Media for BET-therapists (exercise collections)	5

Every component contains a detailed and specific description of each objective and related sub-goals, to appropriate content, the use of behavioural techniques and the interactive education format, on duration, the use of media and combination with other components (see table 3). For the fifteen BET sessions are also short overviews in A3 format available. These represent briefly the relevant components of a current session (see table 4).

Table 3: Example for a component of the fifteen sessions

<table>
<tr><td>BET5_C5
(= BET session 5, component five)</td><td>10 min</td><td>- Flip chart
- TC-BET 23
- TC-BET 44</td></tr>
<tr><td colspan="3">Education: Positive impact of physical activity regarding the interaction of pain and feelings</td></tr>
<tr><td>Aims</td><td colspan="2">- Participants know positive health-related effects of physical activity regarding the interaction of pain and feelings</td></tr>
<tr><td>Content</td><td colspan="2">- Delivery of knowledge about the positive effects of physical activity regarding the interaction of pain and feelings</td></tr>
<tr><td>Method</td><td colspan="2">- Group discussion with the physical therapist as a moderator</td></tr>
<tr><td>BET-technique</td><td colspan="2">► Delivery of Information about the consequences of physical (in-) activity (4)</td></tr>
<tr><td>Execution</td><td colspan="2">Following the components [BET2_C5], [BET4_C5] and [BET5_C3], the connections between stress, mood, muscle tension and pain as well as the positive effects of physical activity are to be discussed.

Delivery of knowledge about the positive effects of physical activity regarding the interaction of pain and feelings

Work out the positive effects of physical activity on the interaction of pain and feelings together with the participants, referring to TC-BET 23 („Pain circle“) and gather the opinions of the participants.

- Distribution of TC-BET 23 („Pain circle“)
- Question for the group:
  ○ How could physical activity influence the „pain circle“?
  ○ Which positive effects did you experience yourself?
- Collection of opinions and exchange in the group
- Summary and provision of basic facts regarding the positive effects of physical activity on the interaction of pain and feelings and the „pain circle“, respectively
  ○ Physical activity increases the release of hormones related to well-being such as serotonin and dopamine; mood and well-being are elevated. One feels fresh, balanced and cheerful.
  ○ Physical activity fosters the release of pain-inhibiting substances. Endorphins reduce pain sensitivity. All in all, the pain threshold is elevated.
  ○ Physical activity improves the blood flow of the muscles and prevents you from having painful tensions!
  ○ Physical activity helps you to distract yourself!
  ○ Regular physical activity improves physical fitness; this is how you get more resilient!
  ○ Less stress hormones are released. Resilience against stress is increased.
- Distribution of TC-BET 44 („Pain circle/circle of well-being“)</td></tr>
</table>

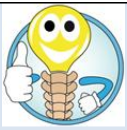	**Key message**<br>○ Physical activity activates and strengthens the resilience regarding pain and the individual „pain-firewall“, respectively. It protects you from the alternating escalation of pain, muscle tension and mood/well-being.
Connections	[BET2_C5], [BET4_C5], [BET5_C3]

(Abbreviations: BET= behavioural exercise therapy, TC = take-home cards, C = component)

Table 4: Example for a short overview of the fifteen sessions

BET Session 5	Components	Materials	Time / Methods
**BET Session 5**	**C1:** Introduction<br>**C2:** Structured active play<br>**C3:** Impact of physical activity on mood and well-being<br>**C4:** Active break<br>**C5:** Impact of physical activity regarding the interaction of pain and feelings<br>**C6:** Resistance training<br>**C7:** Ending	Materials for the therapist:<br>- Exercise mats<br>- Music, if necessary (120-130 BPM)<br><br>Materials for participants:<br>- **TC-BET 21-24**<br>- **TC-BET 12-13**<br>- **TC-BET 44**	**60 min**<br><br>**Methods**

BET5_C1 — **Introduction:** Participant get a review of the last session; possibility for open questions — **5 min**

- Welcoming of participants and review of the last BET session
- Introduction of the content of this session (see above C1-C7)
- Assessment of participants‘ mood
- Information about frequency and extent of health-oriented physical activity referring to **TC-BET 24**

BET5_C2 — **Structured active play:** Participants gain experiences with physical activity and experience pleasure from the cooperation in the mutual game — **5 min**

- **„Shadowing one another“**

BET5_C3 — **Education:** Participants know the positive effects of physical activity on mood and well-being — **10 min**

- Educating participants about the effects of physical activity on mood, well-being and symptoms of stress

Pose open questions to the group and collect opinions of the participants. Include participants collectively with an inductive approach. Ask them, what they already know and refer to their experiences and knowledge. Use simple and pictographic language with a lot of examples and avoid complicated academic terms. Consider that you don't have to address and answer every question at this very moment. The Provision of key messages (see below) is already possible in connection with the discussion about certain themes.

- Summary and provision of key messages about the most important effects of aerobic exercise such as walking, swimming or cycling on mood and well-being with support of **TC-BET 21-22 („Physical activity and well-being“)**

> **Key messages**
> Aerobic exercise such as walking, swimming or cycling, alone or as part of a group strengthen positive aspects of mood such as pleasure, vitality, inner calmness and balance and relaxation. It simply makes you feel good, balanced and cheerful!
>
> Physical activity improves the resilience to stress.

BET5_C4 — **Active break:** Participants experience the positive and refreshing effects of a physically active break — **5 min**

Performance of easy gymnastic exercises

- E.g. walking on tiptoe or heel, lifting knees when walking, making circles with one's shoulders...
- Swing gymnastics: dynamic exercise for the whole body (flexing and extending knees rhythmically in combination with swinging one's arms back and forth; standing position)

BET5_C5 — **Education:** Positive impact of physical activity regarding the interaction of pain and feelings — **10 min**

- Following module [BET5_C3], the interaction of stress with mood, muscle tension and pain as well as the positive effects of physical activity are to be discussed.

Work out the positive effects of physical activity on the interaction of pain and feelings together with the participants, referring to **TC-BET 23 („Pain circle")** and gather the opinions of the participants.

- Distribution of **TC-BET 23 („Pain circle")**
- Question for the group:
  - How could physical activity influence the „pain circle"?
  - Which positive effects did you experience yourself?
- Summary and provision of basic facts regarding the positive effects of physical activity on the interaction of pain and feelings and the „pain circle", respectively.
- Distribution of **TC-BET 44 („Pain circle/circle of well-being")**

> **Key message**
> Physical activity activates and strengthens the resilience regarding pain and the individual „pain-firewall", respectively. It protects you from the alternating escalation of pain, muscle tension and mood/well-being.

VBT5_C6 — **Resistance training – standard program:** Participants perform all muscle-strengthening exercises on their own, rate the intensity on the Borg scale and use their workout plan. — **20 min**

Performing the standard resistance training program

- All exercises from [**List of exercises – resistance training**] are performed in 2-3 sets with 6-20 repetitions in each one. The break between the sets and the exercises should last for about 1 min
- The participants practice on their own
  - Control via subjectively perceived exertion (Borg scale) **[TC-BET 12]**
  - Entry of the number of repetitions and perceived exertion into the workout plan **[TC-BET 13]**
- Correction and assistance when needed (e.g. increase of repetitions, improvement of movement quality, recommendations of how to monitor progress)

VBT5_C7 — **Ending:** Participants reflect on this session and are able to pose open questions — **5 min**

- Conversation to answer open questions
- **Repetition and summary of the key messages**
- Outlook regarding the upcoming session [BET6]

(Abbreviations: BET = behavioural exercise therapy, C = component, TC = take-home cards)

Therapist media and patient cards

Therapist media (e.g. flipcharts) and patient cards support the education on various topics. They are designed with a patient-oriented and challenging character. All cards will be gradually distributed in the BET sessions and additional modules by the BET-therapists and will be collected by the patients in a folder (see cards in figure 2-4 as an example).

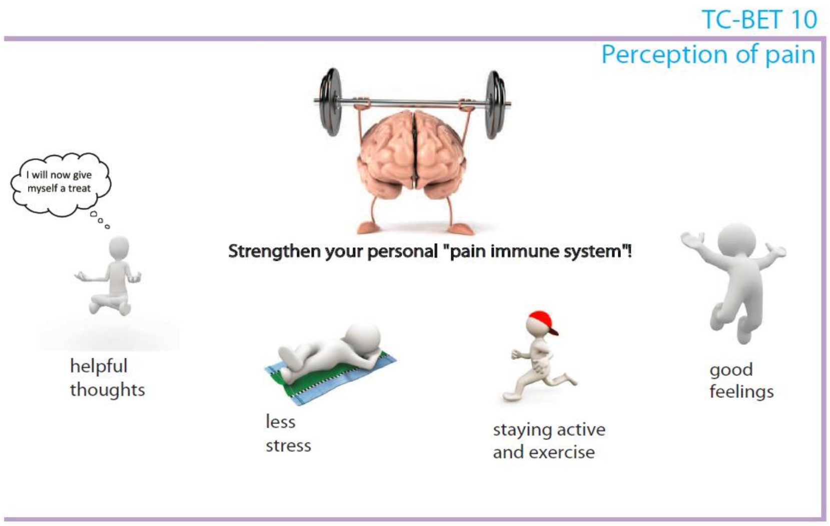

Figure 2: Perception of pain

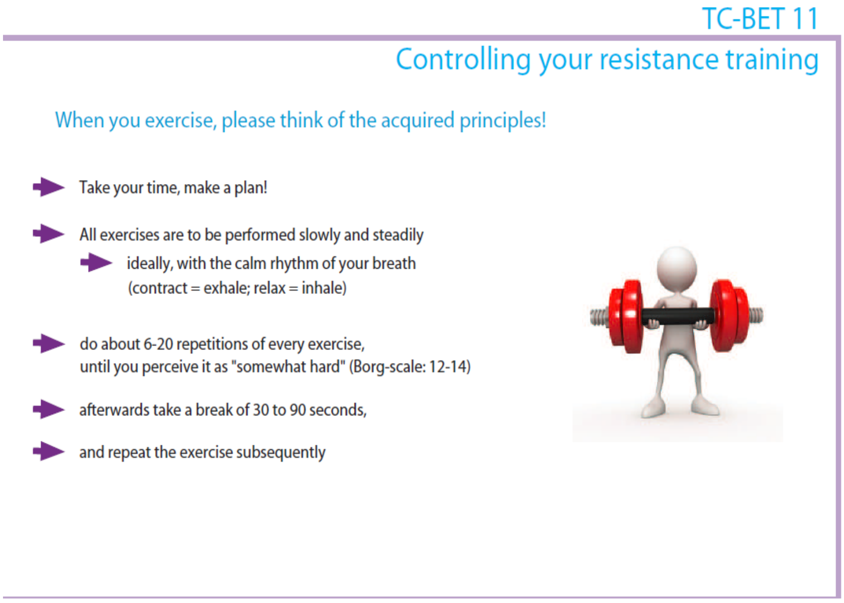

Figure 3: Controlling your resistance training

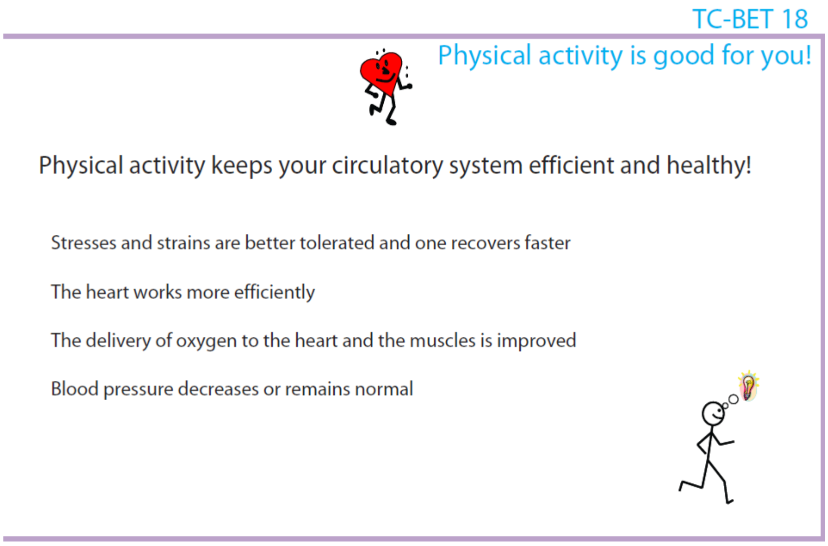

Figure 4: Physical activity is good for you!

References

1. Leeuw M, Goossens MEJB, Linton SJ, Crombez G, Boersma K, Vlaeyen JWS: **The fear-avoidance model of musculoskeletal pain: current state of scientific evidence**. *Journal of behavioral medicine* 2007, **30**:77-94.
2. Hasenbring MI, Verbunt JA: **Fear-avoidance and endurance-related responses to pain: New models of behavior and their consequences for clinical practice**. *Clin. J. Pain* 2010, **26**:747-753.
3. Biddle SJ, Fuchs R: **Exercise psychology: A view from Europe**. *Psychology of Sport and Exercise* 2009, **10**:410-419.
4. Schwarzer R, Lippke S, Luszczynska A: **Mechanisms of health behavior change in persons with chronic illness or disability: The Health Action Process Approach (HAPA)**. *Rehabilitation Psychology* 2011, **56**:161-170.
5. van Middelkoop M, Rubinstein SM, Verhagen AP, Ostelo RW, Koes BW, van Tulder MW: **Exercise therapy for chronic nonspecific low-back pain**. *Best Practice & Research Clinical Rheumatology* 2010, **24**:193-204.
6. Engers A, Jellema P, Wensing M, van der Windt DAWM, Grol R, van Tulder MW: **Individual patient education for low back pain**. *Cochrane Database Syst Rev* 2008:CD004057.
7. Henschke N, Ostelo RW, van Tulder MW, Vlaeyen JW, Morley S, Assendelft WJ, Main CJ: **Behavioural treatment for chronic low-back pain**. *Cochrane Database Syst Rev* 2010, **7**.
8. Verbunt JA, Seelen HA, Vlaeyen JW, van de Heijden GJ, Heuts PH, Pons K, Knottnerus JA: **Disuse and deconditioning in chronic low back pain: concepts and hypotheses on contributing mechanisms**. *European journal of pain (London, England)* 2003, **7**:9-21.
9. Hayden JA, van Tulder MW, Malmivaara A, Koes BW: **Exercise therapy for treatment of non-specific low back pain**. *Cochrane database of systematic reviews (Online)* 2005:CD000335.
10. Macedo LG, Smeets RJEM, Maher CG, Latimer J, McAuley JH: **Graded activity and graded exposure for persistent nonspecific low back pain: A systematic review**. *Phys. Ther* 2010, **90**:860-879.
11. Brox JI, Storheim K, Grotle M, Tveito TH, Indahl A, Eriksen HR: **Evidence-informed management of chronic low back pain with back schools, brief education, and fear-avoidance training**. *Spine J.* 2008, **8**:28-39.
12. Balagué F, Mannion AF, Pellisé F, Cedraschi C: **Non-specific low back pain**. *The Lancet* 2012, **379**:482-491.
13. WHO: **Global recommendations on physical activity for health**. 2010. [http://whqlibdoc.who.int/publications/2010/9789241599979_eng.pdf].

14. Nelson ME, Rejeski WJ, Blair SN, Duncan PW, Judge JO, King AC, Macera CA, Castaneda-Sceppa C: **Physical activity and public health in older adults: recommendation from the American College of Sports Medicine and the American Heart Association**. *Med Sci Sports Exerc* 2007, **39**:1435-1445.
15. Borg GA: **Psychophysical bases of perceived exertion**. *Med Sci Sports Exerc* 1982, **14**:377-381.
16. Geidl W, Hofmann J, Göhner W, Sudeck G, Pfeifer K: **Verhaltensbezogene Bewegungstherapie - Bindung an einen körperlich aktiven Lebensstil**. *Rehabilitation* 2012, **51**:259-268.
17. Conn VS, Hafdahl AR, Brown SA, Brown LM: **Meta-analysis of patient education interventions to increase physical activity among chronically ill adults**. *Patient Educ Couns* 2008, **70**:157-172.
18. Sudeck G, Höner O: **Volitional Interventions within Cardiac Exercise Therapy (VIN-CET): Long-Term Effects on Physical Activity and Health-Related Quality of Life**. *Applied Psychology: Health and Well-Being* 2011, **3**:151-171.
19. Sudeck G: *Motivation und Volition in der Sport- und Bewegungstherapie: Konzeptualisierung und Evaluierung eines Interventionskonzepts zur Förderung von Sportaktivitäten im Alltag.* Hamburg: Czwalina; 2006.
20. Fuchs R, Göhner W, Seelig H, Fleitz A, Mahler C, Schittich I: **Lebensstil-integrierte sportliche Aktivität: Ergebnisse der MoVo-LISA Interventionsstudie**. *B & G* 2010, **26**:270-276.
21. Scholz U, Sniehotta FF: **Langzeiteffekte einer Planungs- und Handlungskontrollintervention auf die körperliche Aktivität von Herzpatienten nach der Rehabilitation**. *Zeitschrift für Gesundheitspsychologie* 2006, **14**:73-81.
