# Supplementary material for: Effects of behavioural exercise therapy on the effectiveness of a multidisciplinary rehabilitation for chronic non-specific low back pain: Study protocol for a randomised controlled trial
Source: BMC Musculoskelet Disord. 2013 Mar 11;14:89. doi: 10.1186/1471-2474-14-89 (PMC3610103; doi:10.1186/1471-2474-14-89)
Supplement: Additional file 3 — BET_Therapy plan. [file 1471-2474-14-89-S3.pdf]

Behavioural exercise therapy (BET) 15 sessions, closed group

sessions/ module	1	2	3	4	5	6	7	8	9	10	11	12	13	14	15
BET closed group 60min	introduction VBT	introduction/review	introduction/review	introduction/review	introduction/review	introduction/review	introduction/review	introduction/review	introduction/review	introduction/review	introduction/review	introduction/review	introduction/review	introduction/review	introduction/review
	activity play - get to know each other	activity play - get to know each other	activity play - get to know each other	activity play - positive exercise experiences	activity play - positive exercise experiences	activity play - positive exercise experiences	activity play - positive exercise experiences	activity play - positive exercise experiences	activity play - positive exercise experiences	activity play - positive exercise experiences	activity play - positive exercise experiences	activity play - positive exercise experiences	activity play - positive exercise experiences	activity play - positive exercise experiences	activity play - positive exercise experiences
	What is low back pain (lbp)? prevalence and course (acute, subacute, chronic lbp)	Why persists my lbp? lack of causes, possibilities and limitations of diagnostic procedures	the spine as a facinating and strong system	effects of physical activity on the organism: „Exercise is good for your body“	effects of physical activity on mood and well-being, stress, mood management	„coper, fear-avoider, endure“ - strengthening pain defense system	physical activity/ exercise as pleasure	education: muscularly stabilization of the spine: How does it work?	education: repetition muscularly stabilization and use of muscularly stabilization during activities of daily life	physical activity in everyday life	repetition: lumbar stabilization exercises during everyday movements	behavior during recurring pain episodes "flare ups"; repetition avoider and endurer	action planning for home: "activity goals and when-where-and how plans"	coping planning for home "barriers and barrier management"	information about aftercare, support in contacting providers
	"exercise break" - swinging gymnastics	"exercise break" - swinging gymnastics	body awareness exercise „human pendulum“	active play ("ball transportation")	"exercise break" - swinging gymnastics		activity play - positive exercise experiences	introduction of lumbar stabilization exercises: activation of deep and global trunk muscles	lumbar stabilization exercises: activation of deep and global trunk muscles during everyday movements	self-directed functional gymnastics *(standard program)*	education: stabilization during work related physical activities				alternative exercises "Qi Gong"
	What are risk factors for lbp? causes and risk factors	Why persists my back pain? consequences of pain, pain memory and positive effects of exercise	self-directed functional gymnastics - introduction of further exercises and training control strategies	dealing with lbp: passive and active self-management strategies	effects of physical activity on the relationship between pain and mood	self-directed functional gymnastics *(standard program)*	thoughts, moods and posture (photo story)	self-directed functional gymnastics *(standard program)*	variation of the body awareness exercise „human pendulum“		one sided loads and compensation strategies	alternative exercises "Qi Gong"			
	one sided loads versus exercise	self-directed functional gymnastics - introduction of further exercises and training control strategies	metaphorical story "mushroom picker"	self-directed functional gymnastics *(standard program)*	self-directed functional gymnastics *(standard program)*		self-directed functional gymnastics *(standard program)*		self-directed functional gymnastics *(standard program)*	alternative exercises "Qi Gong"	alternative exercises "Qi Gong"		alternative exercises "Qi Gong"	alternative exercises "Qi Gong"	final discussion and "take home messages"
	self-directed functional gymnastics (strengthening and stretching exercises) introduction of 2 exercises and training control strategies		relaxation "Swedish short relaxation"			relaxation		relaxation							
	conclusion	conclusion and metaphorical story "Hiking"	conclusion	conclusion	conclusion	conclusion	conclusion	conclusion	conclusion	conclusion	conclusion	conclusion and metaphorical story "Also has all been done?"	conclusion	conclusion	conclusion

Legend colour	Meaning
green	**education about low back pain**
light blue	**exercise/ training/ relaxation**
purple	**coping with low back pain**
yellow	**education about effects of physical activity and exercises**
